# A Call to Re-Evaluate the Role and Responsibility of the Physician in Environmental Health

**DOI:** 10.5696/2156-9614-7.14.1

**Published:** 2017-06-22

**Authors:** Lilian Corra

**Figure:**
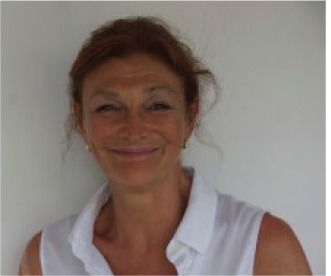


**How is it possible that 25% of children under 5 die from preventable causes? Diseases caused by exposure to the environment are preventable and the responsibility of public health authorities and health professionals can no longer be avoided or ignored.**

In light of recent publications concerning the role of the environment in disease, it is critical to revitalize the commitment to children and adolescents' health and an approach in medicine that prioritizes the “Right to a Healthy Environment.” The environmental and socioeconomic role in disease should return to the forefront of medical thinking and public health policies with a focus on exposure prevention.

Unfortunately, current medical training is focused solely on curing the sick patient. This approach, which does not take into account the environmental factors that cause disease, sacrifices a key opportunity, not only to prevent disease, but also to identify the cause, make the proper diagnosis and implement the appropriate treatment.

The physician's responsibility to determine the proper diagnosis cannot be ignored. Family doctors, pediatricians, obstetricians and other medical professionals are poorly trained to understand the present context of environmental exposure. Throughout the world, the approach and analysis of the patient's environment and factors of socio-environmental risk, which determine the patient's quality of life and eventual illness, are not sufficiently researched or considered.

Without sufficient attention to the familial, social, economic and environmental context (where children and adolescents live, play, grow, learn, etc.), it is impossible to form a complete picture of the possible causes of illness, resulting in a partial and defective analysis. Without a complete and holistic analysis, the diagnosis may be incorrect, and the appropriate treatment and medical advice needed to improve the patient's quality of life or save the patient's life may not be implemented.

Medical consultation should begin with an investigation of the family living environment and background on exposure to environmental risk factors, as well as socio-economic factors, which are all highly significant when analyzing the generation of disease and quality of life of the population and individual.

However, despite being obvious factors when it comes to quality of life and health, in general, these issues are relegated to the periphery. There is practically no systematic record of environmental risk factors (see the WHO Green Page: Assessment of the Environmental Health Risks in Children^1^) in the medical record where environmental factors are specifically investigated and highlighted as a cause of disease and preventative or curative measures are consequently applied.

Currently, medical training in the medical specialty of Health and the Environment enables specialists from this branch of medicine to collaborate with colleagues and examine the socio-economic and environmental context, as well as identify the risk factors or direct causes of a patient's illness, in order to form a diagnosis and implement proper treatment.

The number of cases are increasing in which, with only socio-environmental factors included in the clinical history, the cause of illness can be identified and clinical symptoms reversed by removing the exposure (for example, essential hypertension due to arsenic exposure) or by instituting the appropriate treatment (for example, chelation for adolescents with Guillain Barré syndrome, which is caused by severe mercury poisoning, and in some cases, lead poisoning).

In summary, medical professionals should:

a. – Identify and implement medical education strategies for medical students incorporating the concepts of disease origin and risk factors (including socio-environmental).
  – Complete and reinforce education on the socio-environmental risk factors in the postgraduate medical professional education in a continuous manner.
b. – Implement a holistic approach concerning the patient and the patient's environment.
  – Emphasize the revision of health and disease indicators, adapting them to the 21st Century, adequately addressing updated health concepts through the implementation of health and disease indicators.
c. – Register and introduce environmental factors in the routine approach of prevention and clinical diagnosis.
  – Implement tools planning for health and environmental scenarios to improve the decision-making process in public health, the environment and other areas; Implement tools for the diagnosis of environmental risk factors (WHO Green Page: Assessment of the Environmental Health Risks in Children1) and environmental health clinical history.
d. – Communicate and interact with other sectors.
  – The detection of environmental risk factors demands attention by health professionals in a manner that exceeds the scope of the doctor-patient relationship and strengthens the commitment to health, quality of life and productivity of people and the community.

Adequate communication and reporting by medical professionals to public health authorities of detected environmental risk situations are essential to protect individuals and communities.

The physician has an undeniable role and responsibility to promote and implement effective interventions, which protect the community by both “healing” health ailments and by “remedying” situations where there is risk of environmental contamination and socio-economic decline.

## Debe re-evaluarse el rol y responsabilidades del médico en Salud Ambiental

**Como es posible que un 25% de los niños menores de 5 años mueran por causas evitables? Las enfermedades generadas por la exposición a factores ambientales de riesgo son prevenibles y la responsabilidad, tanto de las autoridades de Salud Pública como de los profesionales de la salud, no puede ser evadida ni ignorada por más tiempo.**

Bajo la luz de las recientes publicaciones sobre carga ambiental de la enfermedad, es necesario revitalizar el compromiso con la salud de los niños y adolescentes en el abordaje de la medicina priorizando el “Derecho a Un Ambiente Sano”. La carga ambiental y socio-económica de la enfermedad debe retomar el centro del pensamiento médico y las políticas de Salud Pública para que se actúe en prevención de la exposición.

Lamentablemente la formación médica hoy en día está centrada en la enfermedad: el paciente enfermo, el diagnóstico y como curarlo. Esta forma de pensamiento médico hace que se pierda la oportunidad de prevenir las enfermedades, detectar la causa, realizar el diagnóstico correcto e implementar el tratamiento adecuado debido a que frecuentemente no se tienen en cuenta los factores ambientales causantes de la enfermedad.

La responsabilidad del médico de llegar al diagnóstico correcto no puede verse diluida. El médico de familia, el pediatra, el obstetra y demás profesionales médicos están hoy en día deficientemente formados para entender el escenario actual de exposición ambiental. En general y en todo el mundo, el abordaje y análisis del entorno de los pacientes y los factores de riesgo ambiental y social que determinan su calidad de vida y eventualmente su enfermedad no son investigados o tenidos en cuenta adecuadamente o en absoluto.

Sin una interpretación adecuada del entorno familiar, social, económico y ambiental (donde los niños y adolescentes viven, crecen, se educan y juegan) no es posible completar el escenario sobre las posibles causas de enfermedad y esto perturba la interpretación y la hace parcial. Sin un análisis holístico y completo, el diagnostico puede ser incorrecto y, por ende, tampoco se puede instaurar el tratamiento adecuado y el consejo médico necesario para mejorar la calidad de vida del paciente o salvar su vida.

La consulta médica debe comenzar con la indagación sobre el ambiente donde la familia vive y los antecedentes sobre exposición a factores ambientales de riesgo, así como los factores socioeconómicos. Ambos factores están íntimamente relacionados cuando se analiza la generación de enfermedades y calidad de vida de la población y de los individuos.

Sin embargo, a pesar de ser temas obvios cuando se habla de calidad de vida y salud, en general son relegados a un segundo plano cuando el medico enfrenta un problema de salud en el paciente. Prácticamente no se implementa un registro sistemático de diagnóstico ambiental (ver Hoja Verde de Diagnóstico Ambiental OMS^1^) en la historia clínica. donde se indague específicamente y se consignen los factores de riesgo ambiental y transparentales para destacarlos como posible causa de enfermedad y aplicar las debidas correcciones preventivas o medidas curativas.

Hoy en día, la formación médica en Salud y Ambiente (Especialidad Médica en Salud y Ambiente) ayuda a que el experto en esta rama de la Medicina colabore con sus colegas para profundizar la conformación del escenario de factores socio-económico-ambientales, identificar los factores de riesgo o causales directas de la enfermedad presentes en la vida del paciente, hacer el diagnóstico certero e implementar el tratamiento correcto.

Se multiplican las publicaciones de casos donde solamente con la inclusión de los factores socio-ambientales en la Historia Clínica se ha llegado a identificar la causa de enfermedad y se ha revertido el cuadro clínico eliminando la exposición (desaparición del cuadro con diagnóstico de hipertensión esencial por arsénico al eliminar la fuente) o instaurando el tratamiento adecuado (quelación de adolescente con síndrome de Guillen Barre por intoxicación grave por mercurio y en otro caso con plomo).

Resumiendo, debemos:

a.- identificar e implementar estrategias de educación médica en los estudiantes de medicina incluyendo tempranamente los conceptos de exposición tóxica temprana desde la concepción y factores de riesgo socio-ambientales;
  - completar y reforzar de manera continua la educación profesional médica de posgrado sobre factores de riesgo socio-ambientales.
b.- Implementar un abordaje holístico del paciente en su ambiente:
  - poner énfasis en revisar los indicadores de salud y enfermedad adecuándolos a los escenarios del SXXI, completando adecuadamente los escenarios de salud actualizados mediante la implementación de indicadores de salud y enfermedad.
c.- Registrar e Introyectar los factores ambientales en la rutina de abordaje de prevención y diagnóstico clínico:
  - implementar las herramientas de diseño de escenarios de salud y ambiente para mejorar el proceso de toma de decisiones en Salud Pública, Ambiente y otras áreas relacionadas;
  - implementar las herramientas de diagnóstico de factores de riesgo ambiental (Hoja Verde de Diagnóstico Ambiental de la OMS^1^) y la historia clínica ambiental.
d.- Comunicar e interactuar con los demás sectores:
  - la detección de los factores ambientales de riesgo merece una toma de acción por parte de los profesionales de la salud que trasciende el ámbito de la relación médicopaciente y fortalece el compromiso con la salud, calidad de vida y productividad de las personas y la comunidad.

La adecuada comunicación y reporte por parte de los profesionales médicos a las autoridades de Salud Pública de las situaciones de riesgo ambiental detectadas es esencial para proteger a los individuos y a las comunidades.

El profesional médico tiene un rol y responsabilidades indeclinables de promover e implementar intervenciones efectivas para actuar en prevención y protección de la comunidad que se ejecutan mediante el acto de “sanar” las afecciones de salud y también al “subsanar” las situaciones de riesgo de contaminación ambiental y deterioro socio/económico.
